# Low vitamin D status and long-term risk of incident fibromyalgia: a propensity score–matched cohort study

**DOI:** 10.3389/fnut.2026.1889243

**Published:** 2026-08-24

**Authors:** Kuo-Chuan Hung, Li-Chen Chang, Kuei-Fen Wang, I-Wen Chen

**Affiliations:** 1 Department of Anesthesiology, Chi Mei Medical Center, Tainan, Taiwan; 2 School of Medicine, College of Medicine, National Sun Yat-sen University, Kaohsiung, Taiwan; 3 Department of Anesthesiology, E-Da Hospital, I-Shou University, Kaohsiung, Taiwan; 4 Department of Anesthesiology, Chi Mei Hospital, Liouying, Tainan, Taiwan

**Keywords:** chronic widespread pain, cohort study, electronic health records, fibromyalgia, propensity score matching, vitamin D deficiency

## Abstract

**Background:**

Cross-sectional studies have reported lower serum vitamin D concentrations in patients with fibromyalgia, but whether vitamin D deficiency (VDD) precedes fibromyalgia onset remains uncertain. We conducted a large retrospective cohort study to evaluate the association between VDD and the long-term risk of incident fibromyalgia.

**Methods:**

Within the TriNetX Network, we identified adults (≥18 years of age) whose serum 25-hydroxyvitamin D was below 20 ng/mL and paired them 1:1 with controls having levels ≥30 ng/mL through propensity score matching. A 1-year landmark period was applied to reduce reverse causation, and outcomes were assessed within an analytic window of up to 10 years. The primary outcome was incident fibromyalgia. The secondary outcome was chronic pain–related diagnoses. All-cause mortality was examined separately as a contextual outcome reflecting overall prognostic burden and the competing risk of death. Sensitivity, sex- and age-stratified subgroup, and severity and chronicity analyses were performed, including severe deficiency defined as 25(OH)D < 10 ng/mL and chronic deficiency defined as two serum 25-hydroxyvitamin D measurements <20 ng/mL recorded within 1 year.

**Results:**

After matching, 561,418 patients were included in both cohorts. VDD conferred an elevated risk of incident fibromyalgia (hazard ratio [HR], 1.63; 95% confidence interval [CI], 1.56–1.71; *p* < 0.001) and chronic pain–related diagnosis (HR, 1.30; 95% CI, 1.28–1.31; *p* < 0.001). All-cause mortality was also higher in the VDD cohort. The association with fibromyalgia was consistent across sensitivity models (HR, 1.35–1.55; all *p* < 0.001), present in both sexes, and stronger among adults > 65 years (HR, 1.76; *p* for subgroup difference <0.001). The severity and chronicity analyses also showed associations with incident fibromyalgia under the severe deficiency definition (HR, 1.89; *p* < 0.001) and the chronic deficiency definition (HR, 1.97; *p* < 0.001).

**Conclusion:**

Low vitamin D status was associated with a higher long-term risk of incident fibromyalgia, with associations also observed under the severe and chronic deficiency definitions. Further prospective studies with repeated vitamin D assessments, detailed pain phenotyping, and mechanistic biomarkers are needed to clarify the clinical and biological relevance of this association.

## 1 Introduction

Fibromyalgia is a chronic pain disorder characterized by widespread musculoskeletal pain, fatigue, sleep disturbances, cognitive dysfunction, and multiple somatic symptoms (1–3). Its prevalence varies according to the diagnostic criteria and study population; however, it is commonly estimated to affect approximately 2–4% of the general population (4–7). Fibromyalgia substantially impairs physical function and quality of life and is associated with increased healthcare utilization (8, 9). However, the upstream biological and clinical factors that predispose individuals to its development remain incompletely understood (10–13). Current concepts emphasize fibromyalgia as a disorder of altered pain processing, in which central sensitization, neuroinflammatory and immune activation, impaired descending pain modulation, and altered peripheral sensory input may contribute to the persistent pain amplification (13–15). Because widespread pain is a defining feature of fibromyalgia, chronic widespread pain has often been considered a broader clinical pain phenotype that overlaps with or may precede fibromyalgia within the spectrum of persistent pain amplification (16, 17).

Clinical evidence also suggests a possible relationship between vitamin D status and fibromyalgia-related pain phenotypes. Previous meta-analyses have reported lower serum vitamin D concentrations in patients with fibromyalgia than in controls (18, 19). In line with these observations, a meta-analysis of chronic widespread pain, a broader fibromyalgia-related pain phenotype, found that hypovitaminosis D was associated with higher odds of chronic widespread pain, with a pooled adjusted odds ratio of 1.41 (20). More recent reviews of vitamin D supplementation in fibromyalgia have suggested potential improvements in fibromyalgia-related impact or pain-related outcomes, although the available trials remain limited by small sample sizes, heterogeneity in dosing regimens, and variable outcome definitions (21–23). Thus, while there is mechanistic rationale and preliminary clinical data, the directional link between vitamin D deficiency (VDD) and later fibromyalgia development has yet to be firmly established. To fill this knowledge gap, we leveraged the TriNetX Global Collaborative Network to perform a sizeable retrospective cohort analysis assessing the link between low vitamin D status and long-term fibromyalgia incidence.

## 2 Methods

### 2.1 Data source and ethical statement

We performed this retrospective cohort analysis on the TriNetX Global Collaborative Network platform, a federated electronic health record research environment offering longitudinal, de-identified clinical records contributed by participating healthcare organizations. Because investigators had no access to directly identifiable patient data, the requirement for informed consent was waived. Institutional Review Board approval for the protocol was granted by Chi Mei Medical Center (Tainan, Taiwan). The work adhered to the principles of the Declaration of Helsinki and applicable institutional standards. Study data were extracted on May 9, 2026. Complete cohort definitions, temporal query logic, and all diagnostic, laboratory, medication, encounter, and procedural codes used for cohort construction, exclusion criteria, propensity score matching, and outcome ascertainment are provided in Supplementary Table 1.

### 2.2 Exposure definition

Eligible adult patients (≥18 years) in both the VDD and control cohorts were required to have at least one serum 25-hydroxyvitamin D measurement recorded between January 1, 2010, and December 31, 2024, and at least two documented healthcare encounters within the 5 years preceding the index date to ensure active engagement with the healthcare system and reliable ascertainment of baseline comorbidities. Patients with a serum 25-hydroxyvitamin D concentration <20 ng/mL (measured as calcidiol or calcidiol/ercalcidiol) were categorized as the VDD cohort, while those with concentrations ≥30 ng/mL constituted the control cohort. Patients with intermediate concentrations of 20.0–29.9 ng/mL were not included because the study was designed to compare clinically defined VDD with clearly normal vitamin D status. For every patient, the date of the first measurement meeting the eligibility criteria served as the index date. To improve exposure specificity, patients in the VDD cohort were excluded if any 25-hydroxyvitamin D value ≥20 ng/mL was recorded within the 5-year look-back period, and control patients were excluded if any value <30 ng/mL was recorded during the same period (Supplementary Table 1).

### 2.3 Exclusion criteria

Several categories of patients were excluded from both cohorts to improve the ascertainment of incident outcomes and reduce confounding. First, because fibromyalgia, other chronic pain, and chronic pain syndrome may overlap clinically or may be coded before a formal diagnosis of fibromyalgia, patients with any recorded diagnosis of fibromyalgia (ICD-10-CM: M79.7), other chronic pain (G89.29), or chronic pain syndrome (G89.4) at any time in the available EHR history before the index date were excluded from the study. Second, we excluded patients with conditions that may independently cause widespread pain or confound the association between vitamin D status and pain-related outcomes, including rheumatoid arthritis (M05, M06), systemic lupus erythematosus (M32), dermatomyositis (M33), opioid-related disorders (F11), bipolar disorder (F31), and schizophrenia spectrum disorders (F20–F29). Third, patients with conditions that may substantially affect vitamin D metabolism or absorption were excluded, including osteomalacia (M83), celiac disease (K90.0), inflammatory bowel disease (K50, K51), postsurgical malabsorption (K91.2), hepatic fibrosis or cirrhosis (K74), end-stage renal disease, stage 4–5 chronic kidney disease, dialysis dependence, and prior bariatric surgery. The complete diagnostic and procedural code definitions used for these exclusions are provided in Supplementary Table 1.

### 2.4 Landmark analysis

A 1-year landmark design was applied to reduce reverse causation and improve incident outcome ascertainment. Subjects were excluded if death, fibromyalgia, other chronic pain conditions, or chronic pain syndrome occurred within the one-year period after the index date. Follow-up began 365 days after the index date and continued until the first outcome event, death, last recorded encounter, or 3,650 days after the index date, whichever occurred first. Accordingly, the study used an analytic window of up to 10 years rather than requiring 10 years of follow-up for every patient. Osteoporosis with current pathological fracture was additionally excluded before and during the landmark period to ensure incident ascertainment of this positive control outcome.

### 2.5 Propensity score matching

To enhance comparability across the two cohorts, we applied 1:1 propensity score matching via a greedy nearest-neighbor algorithm with a caliper set at 0.1 pooled standard deviations. Propensity scores were estimated using prespecified covariates, including demographic characteristics, cardiometabolic and vascular comorbidities, renal and hepatic diseases, neuropsychiatric disorders, nutritional and inflammatory conditions, medication exposure, and available laboratory indicators (Supplementary Table 1). Covariate balance was evaluated using standardized mean differences, with values <0.10 considered acceptable. Propensity score density plots were inspected before and after matching to evaluate cohort overlaps. Although the measurement date was used internally by the TriNetX platform to establish the index date, the calendar month or season of individual measurements was not available in the aggregated analytic output and therefore could not be incorporated into propensity score matching or outcome analyses.

### 2.6 Primary, secondary, and control outcomes

The primary outcome was incident fibromyalgia (ICD-10-CM code: M79.7). The secondary outcomes included chronic pain-related diagnoses (ICD-10-CM: G89.29, G89.4; Supplementary Table 1). Chronic pain–related diagnoses were included as a secondary outcome because fibromyalgia shares substantial clinical overlap with other persistent pain conditions, and evaluating this broader outcome may capture cases in which widespread pain or fibromyalgia-related symptoms were coded under alternative pain diagnoses. All-cause mortality was examined separately as a contextual outcome reflecting overall prognostic burden and the competing risk of death, rather than as corroborative evidence for the association between VDD and fibromyalgia. Positive control outcomes included osteoporotic fractures (ICD-10-CM: M80), low uncorrected total serum calcium (<8.5 mg/dL), and secondary hyperparathyroidism (ICD-10-CM: N25.81), which are established sequelae of VDD and were assessed to support the internal validity of the exposure definition. Albumin-corrected calcium could not be derived because it was not available as an analyzable variable within the TriNetX platform. Acute appendicitis (ICD-10-CM code: K35) was used as a negative control outcome. Healthcare utilization was assessed using the available binary indicator of at least one healthcare encounter during follow-up. The aggregated analytic output did not provide the clinical indications for vitamin D testing or more granular utilization measures, including total encounter counts, visit frequency per person-year, specialty-specific contacts, or pain-related diagnostic encounters.

### 2.7 Sensitivity, subgroup, and multivariable analyses

Three prespecified sensitivity analyses were conducted to evaluate the robustness of the primary findings. Each sensitivity analysis was implemented as a separate analysis-specific TriNetX query, with propensity scores re-estimated and 1:1 matching repeated within the eligible population using the same covariates, greedy nearest-neighbor algorithm, and caliper as in the primary analysis. Accordingly, the resulting sample sizes represent independently matched cohorts rather than subsets obtained by directly filtering the primary matched population. Model I restricted the study period to 2016–2024 to assess whether the association was consistent when limited to more recent data with potentially improved coding practices. Model II excluded patients who died during follow-up to determine whether the observed association was driven by the differential mortality. Model III was restricted to patients with at least one recorded healthcare encounter during follow-up to minimize the influence of loss to follow-up or incomplete outcome data. Subgroup analyses were stratified by sex (male vs. female) and age group (18–65 vs. >65 years), with each stratum constructed as a separate TriNetX query and independently matched using the same propensity score–matching procedure. Because each subgroup was constructed and matched separately, between-subgroup differences were evaluated outside the TriNetX platform using a two-sided z test based on the unrounded log hazard ratios and their standard errors. These comparisons were exploratory and did not represent formal interaction tests based on a product term within a common Cox model. Sequential multivariable Cox models were fitted with cohort membership as the main exposure, progressively adjusted for demographics, cardiometabolic risk factors, pain-related and neuropsychiatric comorbidities, and other medical conditions.

### 2.8 Severity and chronicity analyses

We carried out two additional analyses to evaluate the associations of VDD severity and repeated documentation with incident fibromyalgia. First, patients with severe VDD, defined as serum 25-hydroxyvitamin D < 10 ng/mL, were compared with controls with normal vitamin D status (≥30 ng/mL) to assess whether a lower exposure threshold yielded a larger effect size. Second, chronic VDD was defined as two serum 25-hydroxyvitamin D measurements <20 ng/mL recorded within 1 year, whereas normal vitamin D status in the control group was defined as two serum 25-hydroxyvitamin D measurements ≥30 ng/mL recorded within 1 year. The index date was defined as the date of the second qualifying measurement. This analysis aimed to identify patients with repeatedly documented VDD rather than a single transient low value, thereby reducing exposure misclassification without constituting a formal trend analysis across vitamin D categories.

### 2.9 Statistical analysis

Time-to-event outcomes were analyzed using Cox proportional hazards models, and the results are reported as hazard ratios (HRs) and 95% confidence intervals (CIs). The primary analysis adopted a cause-specific modeling framework because the objective was to estimate the association between VDD and fibromyalgia occurrence rather than to estimate the absolute cumulative incidence under competing risks. To account for the competing event of mortality, a competing risk analysis using the Aalen–Johansen estimator was additionally performed in the full unmatched cohort to estimate the cumulative incidence of fibromyalgia while treating death as a competing event. The TriNetX analytic platform did not support application of the Aalen–Johansen estimator within the propensity score–matched cohort; therefore, this analysis was limited to the full unmatched cohort and was interpreted as a supplementary descriptive analysis rather than as a replacement for the primary matched cause-specific Cox analysis. The assumption of proportional hazards was evaluated by means of Schoenfeld residuals. A two-sided *α* of 0.05 defined statistical significance for the primary analysis. E-values were calculated for the primary HR and the lower limit of its 95% CI to assess robustness to potential unmeasured confounding. Other outcomes such as control outcomes and subgroup models were treated as exploratory, so no correction for multiple comparisons was applied. Missing data were not imputed. To make measurement availability explicit, laboratory covariates were additionally categorized according to prespecified thresholds or as not measured in Supplementary Table 2. Percentages were calculated using the full matched cohort as the denominator.

## 3 Results

### 3.1 Patient selection and baseline characteristics

**Figure 1:**
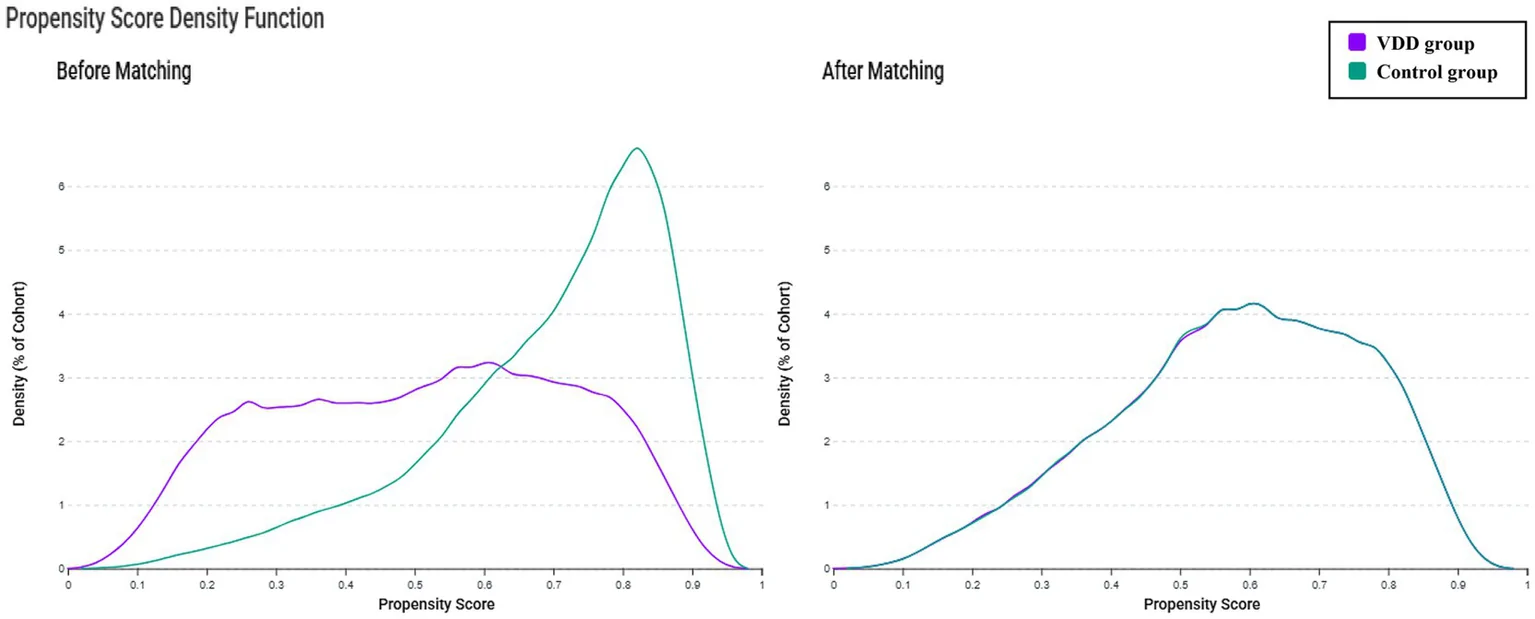
Propensity score density distributions before and after matching. Propensity score density distributions are shown for patients with vitamin D deficiency (VDD) and controls with normal vitamin D status (control group) before and after 1:1 propensity score matching. Improved overlap after matching indicates better covariate balance between groups. VDD was defined as serum 25(OH)D < 20 ng/mL, and normal vitamin D status was defined as ≥30 ng/mL.

A total of 722,662 patients met the criteria for the VDD cohort and 1,264,612 for the control cohort before matching. Following matching, each cohort retained 561,418 patients. Before matching, substantial differences were observed between the cohorts, particularly in age (46.1 ± 20.5 vs. 55.9 ± 19.1 years) and racial composition (White: 47.6% vs. 76.4%). After matching, all standardized mean differences were reduced to below 0.10 (Table 1). Propensity score density plots confirmed an improved distributional overlap between the cohorts after matching (Figure 1). In the matched cohorts, the mean age was approximately 50 years, approximately 66.5% were female, and the prevalent comorbidities included hypertension (approximately 28%), dyslipidemia (approximately 27%), and neoplasms (approximately 19%). The mean follow-up duration was 1,853.6 ± 1,144.0 days in the VDD cohort and 1,753.4 ± 1,118.1 days in the control cohort; the corresponding median follow-up durations were 1,699 and 1,538 days, respectively.

**Table 1:** Baseline characteristics of patients with low vitamin D status and matched controls before and after propensity score matching.

Variables	Before matching			After matching		
	VDD group (*n* = 722,662)	Control group (*n* = 1,264,612)	SMD	VDD group (*n* = 561,418)	Control group (*n* = 561,418)	SMD
Patient characteristics						
Age at index (years)	46.1 ± 20.5	55.9 ± 19.1	0.498	49.5 ± 20.0	50.0 ± 20.0	0.023
BMI ≥ 30 (kg/m^2^)	230,096 (31.8)	294,758 (23.3)	0.192	160,934 (28.7)	164,713 (29.3)	0.015
Female	473,814 (65.6)	896,358 (70.9)	0.114	374,467 (66.7)	372,640 (66.4)	0.007
White	343,660 (47.6)	966,077 (76.4)	0.622	328,475 (58.5)	325,121 (57.9)	0.012
Black or African American	172,288 (23.8)	95,629 (7.6)	0.459	82,967 (14.8)	85,295 (15.2)	0.012
Hispanic or Latino	74,976 (10.4)	43,985 (3.5)	0.274	38,999 (6.9)	37,191 (6.6)	0.013
Asian	29,911 (4.1)	47,574 (3.8)	0.019	26,487 (4.7)	25,432 (4.5)	0.009
Comorbidities and healthcare utilization						
Essential (primary) hypertension	193,602 (26.8)	395,055 (31.2)	0.098	154,429 (27.5)	157,735 (28.1)	0.013
Encounter for general adult medical examination†	184,075 (25.5)	398,746 (31.5)	0.135	151,515 (27.0)	153,535 (27.3)	0.008
Disorders of lipoprotein metabolism and other lipidemias	176,972 (24.5)	437,720 (34.6)	0.223	150,609 (26.8)	153,958 (27.4)	0.013
Neoplasms	123,100 (17.0)	304,137 (24.1)	0.174	105,740 (18.8)	106,531 (19.0)	0.004
Anxiety, dissociative, stress-related, somatoform and other nonpsychotic mental disorders	121,934 (16.9)	218,894 (17.3)	0.012	96,404 (17.2)	97,174 (17.3)	0.004
Other nutritional deficiencies	104,070 (14.4)	258,315 (20.4)	0.159	87,655 (15.6)	89,747 (16.0)	0.010
Dorsalgia	112,627 (15.6)	192,673 (15.2)	0.010	84,627 (15.1)	85,106 (15.2)	0.002
Diabetes mellitus	92,076 (12.7)	144,096 (11.4)	0.041	68,226 (12.2)	69,128 (12.3)	0.005
Disorders of thyroid gland	75,147 (10.4)	210,593 (16.7)	0.184	66,082 (11.8)	67,093 (12.0)	0.006
Sleep disorders	79,913 (11.1)	142,175 (11.2)	0.006	60,903 (10.8)	61,112 (10.9)	0.001
Osteoarthritis	57,253 (7.9)	149,854 (11.9)	0.132	49,464 (8.8)	50,248 (9.0)	0.005
Nicotine dependence	58,474 (8.1)	57,496 (4.5)	0.146	37,546 (6.7)	37,696 (6.7)	0.001
Ischemic heart diseases	44,200 (6.1)	96,225 (7.6)	0.059	36,661 (6.5)	37,124 (6.6)	0.003
Migraine	34,837 (4.8)	56,178 (4.4)	0.018	26,177 (4.7)	26,165 (4.7)	0.000
Chronic kidney disease (CKD)	28,722 (4.0)	63,711 (5.0)	0.051	23,297 (4.2)	24,097 (4.3)	0.007
Iron deficiency anemia	33,052 (4.6)	43,701 (3.5)	0.057	22,282 (4.0)	22,597 (4.0)	0.003
Cerebrovascular diseases	27,373 (3.8)	55,011 (4.4)	0.028	21,967 (3.9)	22,264 (4.0)	0.003
Diseases of liver	28,080 (3.9)	43,053 (3.4)	0.026	20,924 (3.7)	20,815 (3.7)	0.001
COVID-19	26,780 (3.7)	47,378 (3.7)	0.002	20,667 (3.7)	20,667 (3.7)	0.000
COPD	22,796 (3.2)	37,868 (3.0)	0.009	17,853 (3.2)	17,950 (3.2)	0.001
Heart failure	24,268 (3.4)	35,934 (2.8)	0.030	17,635 (3.1)	17,641 (3.1)	0.000
Depressive disorder	21,249 (2.9)	31,447 (2.5)	0.028	15,950 (2.8)	15,912 (2.8)	0.000
Alcohol related disorders	16,881 (2.3)	17,136 (1.4)	0.073	10,671 (1.9)	10,552 (1.9)	0.002
Chronic fatigue	6,811 (0.9)	16,800 (1.3)	0.036	5,848 (1.0)	5,980 (1.1)	0.002
Malnutrition	7,435 (1.0)	10,196 (0.8)	0.023	5,387 (1.0)	5,461 (1.0)	0.001
Systemic connective tissue disorders	4,397 (0.6)	13,627 (1.1)	0.051	3,877 (0.7)	4,039 (0.7)	0.003
Reduced mobility	3,577 (0.5)	5,715 (0.5)	0.006	2,650 (0.5)	2,671 (0.5)	0.001
Laboratory data						
Hemoglobin ≥ 12 g/dL	464,941 (64.3)	760,152 (60.1)	0.087	360,806 (64.3)	367,562 (65.5)	0.025
Albumin ≤ 3.5 g/dL	93,300 (12.9)	111,247 (8.8)	0.133	62,935 (11.2)	64,659 (11.5)	0.010
HbA1c ≥ 9%	33,958 (4.7)	26,065 (2.1)	0.146	19,775 (3.5)	19,437 (3.5)	0.003
eGFR ≤60 mL/min/1.73 m^2^	89,364 (12.4)	157,942 (12.5)	0.004	70,674 (12.6)	74,043 (13.2)	0.018
C-reactive protein ≥ 10 mg/L	46,865 (6.5)	42,459 (3.4)	0.145	30,148 (5.4)	29,433 (5.2)	0.006
Medications						
Corticosteroids for systemic use	231,714 (32.1)	410,371 (32.5)	0.008	176,045 (31.4)	178,936 (31.9)	0.011
Opioid analgesics	224,658 (31.1)	371,843 (29.4)	0.037	168,274 (30.0)	170,245 (30.3)	0.008
Benzodiazepine	152,216 (21.1)	290,876 (23.0)	0.047	120,254 (21.4)	121,608 (21.7)	0.006
Antidepressants	129,723 (18.0)	246,640 (19.5)	0.040	105,261 (18.7)	106,393 (19.0)	0.005
Blood glucose lowering drugs, excl. Insulins	67,635 (9.4)	105,603 (8.4)	0.036	50,096 (8.9)	50,827 (9.1)	0.005
Vitamin d supplementation	50,476 (7.0)	153,885 (12.2)	0.177	43,632 (7.8)	44,976 (8.0)	0.009
Insulins and analogues	56,286 (7.8)	67,458 (5.3)	0.099	38,058 (6.8)	38,066 (6.8)	0.000
Gabapentinoids	43,668 (6.0)	81,974 (6.5)	0.018	34,035 (6.1)	34,437 (6.1)	0.003

Data are presented as *n* (%) or mean ± standard deviation. Standardized mean differences <0.10 were considered acceptable for covariate balance. BMI, body mass index; CKD, chronic kidney disease; COPD, chronic obstructive pulmonary disease; eGFR, estimated glomerular filtration rate; HbA1c, hemoglobin A1c; SMD, standardized mean difference; VDD, vitamin D deficiency. ^†^The encounter for general adult medical examination was treated as a baseline covariate and did not indicate that the index 25(OH)D measurement was obtained during that examination.

### 3.2 Primary and secondary outcomes

After the 1-year landmark period, 4,771 (0.85%) patients in the VDD cohort and 2,745 (0.49%) in the control cohort developed incident fibromyalgia, corresponding to an absolute difference in observed event proportions of 0.36 percentage points. Compared with normal vitamin D status, low vitamin D status was associated with a higher long-term risk of incident fibromyalgia (HR, 1.63; 95% CI, 1.56–1.71; *p* < 0.001; Table 2). The proportionality test based on Schoenfeld residuals showed no evidence that the proportional hazards assumption was violated (*p* = 0.699). Among the secondary outcomes, low vitamin D status was also associated with a higher risk of chronic pain-related diagnoses (HR, 1.30; *p* < 0.001). All-cause mortality, assessed as a contextual outcome, was also higher in the VDD cohort (HR, 1.43; *p* < 0.001). The E-values for the primary HR and its lower confidence limit were 2.64 and 2.49, respectively. Regarding control outcomes, the positive controls of secondary hyperparathyroidism (HR, 2.14; *p* < 0.001), low uncorrected total serum calcium (HR, 1.26; *p* < 0.001), and osteoporotic fracture (HR, 1.40; *p* < 0.001) all showed significant associations with low vitamin D status, supporting the validity of exposure classification. The negative control, appendicitis, demonstrated no significant association (HR, 1.04; 95% CI, 0.98–1.11; *p* = 0.155). The proportion of patients with at least one healthcare visit during follow-up was numerically similar between cohorts (91.17% vs. 91.75%), although the difference was statistically significant because of the large sample size.

**Table 2:** Association between low vitamin D status and incident fibromyalgia, chronic pain-related diagnoses, all-cause mortality, and control outcomes after propensity score matching.

Outcome	VDD group (*n* = 561,418)	Control group (*n* = 561,418)	HR (95% CI)	*p* value
	Events (%)	Events (%)		
Primary outcome				
Fibromyalgia	4,771 (0.85)	2,745 (0.49)	1.63 (1.56–1.71)	<0.001
Secondary outcomes				
Chronic pain-related diagnosis	60,859 (10.84)	44,945 (8.01)	1.30 (1.28–1.31)	<0.001
Contextual outcome				
Mortality	31,479 (5.61)	20,488 (3.65)	1.43 (1.41–1.46)	<0.001
Positive control outcome				
Secondary Hyperparathyroidism	1,051 (0.19)	463 (0.08)	2.14 (1.92–2.39)	<0.001
Hypocalcemia	79,299 (14.13)	61,030 (10.87)	1.26 (1.24–1.27)	<0.001
Osteoporotic fracture	2,474 (0.44)	1,628 (0.29)	1.40 (1.32–1.49)	<0.001
Negative control outcome				
Appendicitis	2,361 (0.42)	2,116 (0.38)	1.04 (0.98–1.11)	0.155
Healthcare utilization				
At least one healthcare visit during follow-up	511,835 (91.17)	515,082 (91.75)	0.90 (0.90–0.90)	<0.001

Data are presented as number of events (%). Chronic pain-related diagnoses included ICD-10-CM codes G89.29 and G89.4. CI, confidence interval; HR, hazard ratio; VDD, vitamin D deficiency. Hypocalcemia was defined as uncorrected total serum calcium <8.5 mg/dL.

### 3.3 Sensitivity analyses and subgroup analyses

Across all three sensitivity analyses, the association between VDD and incident fibromyalgia remained statistically significant, although the estimate was attenuated in Model I (Table 3). When the study period was restricted to 2016–2024 (Model I), the HR was 1.35 (*p* < 0.001). After excluding patients who died during follow-up (Model II), the HR was 1.55 (*p* < 0.001). When restricted to patients with at least one follow-up encounter (Model III), the HR was 1.54 (*p* < 0.001). Associations with secondary and positive control outcomes were similarly consistent across all models. Appendicitis was modestly associated with VDD in Model I (HR, 1.13; 95% CI, 1.05–1.21; *p* = 0.001), but not in Models II (HR, 1.04; *p* = 0.164) or III (HR, 1.04; *p* = 0.181; Table 3).

**Table 3:** Sensitivity analyses of the association between low vitamin D status and risk of incident fibromyalgia.

Outcomes	Model I (*n* = 139,486 for each group)		Model II (*n* = 137,723 for each group)		Model III (*n* = 107,871 for each group)	
	HR (95% CI)	*p* value	HR (95% CI)	*p* value	HR (95% CI)	*p* value
Fibromyalgia	1.35 (1.27–1.42)	<0.001	1.55 (1.48–1.62)	<0.001	1.54 (1.48–1.60)	<0.001
Chronic pain-related diagnosis	1.27 (1.25–1.29)	<0.001	1.29 (1.28–1.31)	<0.001	1.28 (1.26–1.29)	<0.001
Mortality	1.53 (1.50–1.56)	<0.001	na	na	1.44 (1.42–1.46)	<0.001
Hyperparathyroidism	1.98 (1.75–2.25)	<0.001	2.00 (1.78–2.25)	<0.001	2.10 (1.89–2.34)	<0.001
Hypocalcemia	1.25 (1.24–1.27)	<0.001	1.25 (1.24–1.27)	<0.001	1.27 (1.26–1.29)	<0.001
Osteoporotic fracture	1.27 (1.19–1.37)	<0.001	1.47 (1.37–1.57)	<0.001	1.43 (1.34–1.52)	<0.001
Appendicitis	1.13 (1.05–1.21)	0.001	1.04 (0.98–1.11)	0.164	1.04 (0.98–1.10)	0.181
At least one healthcare visit during follow-up	0.89 (0.89–0.90)	<0.001	0.90 (0.90–0.91)	<0.001	0.91 (0.90–0.91)	<0.001

Model I restricted the study period to 2016–2024. Model II excluded patients who died during follow-up. Model III was restricted to patients with at least one recorded healthcare encounter during follow-up. Outcome ascertainment began after the 1-year landmark period. Chronic pain-related diagnoses included ICD-10-CM codes G89.29 and G89.4. CI, confidence interval; HR, hazard ratio. Na: data not available. Each analysis was conducted as a separate TriNetX query and independently matched using the same propensity score–matching procedure as the primary analysis. All included covariates achieved a post-matching SMD <0.10. Hypocalcemia was defined as uncorrected total serum calcium <8.5 mg/dL.

Sex-stratified analyses showed that the association with incident fibromyalgia was present in both men (HR, 1.42; *p* < 0.001) and women (HR, 1.56; *p* < 0.001), with no statistically significant difference between the subgroup-specific estimates (p for subgroup difference = 0.097; Table 4). In age-stratified analyses (Table 5), the association was present in both the 18–65 years group (HR, 1.45; *p* < 0.001) and the >65 years group (HR, 1.76; *p* < 0.001), with a significant between-subgroup difference indicating a stronger association among older adults (*p* for subgroup difference <0.001).

**Table 4:** Sex-stratified subgroup analyses of the association between low vitamin D status and risk of incident fibromyalgia.

Outcomes	Male (*n* = 197,029 for each group)		Female (*n* = 381,882 for each group)		*p* for subgroup difference
	HR (95% CI)	*p* value	HR (95% CI)	*p* value	
Fibromyalgia	1.42 (1.28–1.58)	<0.001	1.56 (1.49–1.63)	<0.001	0.097
Chronic pain-related diagnosis	1.21 (1.18–1.23)	<0.001	1.32 (1.30–1.34)	<0.001	<0.001
Mortality	1.42 (1.39–1.46)	<0.001	1.47 (1.44–1.50)	<0.001	0.034

Chronic pain-related diagnoses included ICD-10-CM codes G89.29 and G89.4. CI, confidence interval; HR, hazard ratio. Each analysis was conducted as a separate TriNetX query and independently matched using the same propensity score–matching procedure as the primary analysis. All included covariates achieved a post-matching SMD <0.10.

**Table 5:** Age-stratified subgroup analyses of the association between low vitamin D status and risk of incident fibromyalgia.

Outcomes	18–65 years (*n* = 359,263 for each group)		>65 years (*n* = 216,913 for each group)		*p* for subgroup difference
	HR (95% CI)	*p* value	HR (95% CI)	*p* value	
Fibromyalgia	1.45 (1.37–1.52)	<0.001	1.76 (1.65–1.88)	<0.001	<0.001
Chronic pain-related diagnosis	1.24 (1.22–1.26)	<0.001	1.36 (1.33–1.38)	<0.001	<0.001
Mortality	1.37 (1.31–1.43)	<0.001	1.43 (1.41–1.46)	<0.001	0.070

Chronic pain-related diagnoses included ICD-10-CM codes G89.29 and G89.4. CI, confidence interval; HR, hazard ratio. Each analysis was conducted as a separate TriNetX query and independently matched using the same propensity score–matching procedure as the primary analysis. All included covariates achieved a post-matching SMD <0.10.

### 3.4 Sequential multivariable cox regression

The association between VDD and incident fibromyalgia persisted across the progressively adjusted Cox regression models (Table 6). The HR ranged from 1.74 (95% CI, 1.69–1.80) in the model adjusted for age, sex, and race/ethnicity to 1.67 (95% CI, 1.61–1.72) in the fully adjusted model incorporating demographics, cardiometabolic factors, pain-related and neuropsychiatric comorbidities, and other medical conditions. The minimal attenuation across sequential models suggests that the observed association was not substantially driven by the measured confounders.

**Table 6:** Sequential multivariable Cox regression models for the association between low vitamin D status and incident fibromyalgia.

Model	Matching/Adjustment variables included	HR (95% CI)	*p* value
Model 1	Age at index, sex, race/ethnicity	1.74 (1.69–1.80)	<0.001
Model 2	Model 1 + diabetes mellitus, dyslipidemia, nicotine dependence, alcohol-related disorders	1.71 (1.66–1.77)	<0.001
Model 3	Model 2 + dorsalgia, sleep disorders, osteoarthritis, migraine, recurrent major depressive disorder, pain not elsewhere classified, chronic fatigue	1.68 (1.62–1.74)	<0.001
Model 4	Model 3 + hypertension, neoplasms, other nutritional deficiencies, thyroid disorders, CKD, cerebrovascular disease, liver disease, malnutrition, systemic connective tissue disorders	1.67 (1.61–1.72)	<0.001

Sequential multivariable Cox regression analyses were performed in the unmatched cohort to assess the robustness of the association between low vitamin D status and incident fibromyalgia after progressive covariate adjustment. CI, confidence interval; HR, hazard ratio.

### 3.5 Severity and chronicity analyses

The severity and chronicity analyses showed associations under more stringent definitions of VDD (Tables 7, 8). In the severe VDD analysis, the association with incident fibromyalgia was numerically larger (HR, 1.89; *p* < 0.001) than the estimate observed in the primary analysis (HR, 1.63). Because these estimates were derived from separately constructed and matched cohorts, this comparison should be interpreted descriptively. The chronic VDD analysis also showed an association with incident fibromyalgia (HR, 1.97; p < 0.001). Associations with chronic pain-related diagnoses, mortality, and positive control outcomes were also observed in both analyses, whereas appendicitis remained non-significant.

**Table 7:** Association between severe Vitamin D deficiency and risk of incident fibromyalgia.

Outcome	Severe VDD group (*n* = 200,909)	Control group (*n* = 200,909)	HR (95% CI)	*p* value
	Events (%)	Events (%)		
Fibromyalgia	2,450 (1.22)	1,164 (0.58)	1.89 (1.76–2.02)	<0.001
Chronic pain-related diagnosis	24,728 (12.31)	16,653 (8.29)	1.38 (1.35–1.40)	<0.001
Mortality	14,775 (7.35)	8,853 (4.41)	1.50 (1.46–1.54)	<0.001
Hyperparathyroidism	675 (0.34)	197 (0.10)	3.13 (2.67–3.66)	<0.001
Hypocalcemia	35,914 (17.88)	24,355 (12.12)	1.42 (1.39–1.44)	<0.001
Osteoporotic fracture	1,027 (0.51)	581 (0.29)	1.54 (1.39–1.70)	<0.001
Appendicitis	830 (0.41)	702 (0.35)	1.06 (0.96–1.18)	0.224
At least one healthcare visit during follow-up	182,341 (90.76)	183,605 (91.39)	0.92 (0.92–0.93)	<0.001

Severe VDD was defined as serum 25(OH)D < 10 ng/mL; controls had normal vitamin D status, defined as serum 25(OH)D ≥ 30 ng/mL. Data are presented as number of events (%). Chronic pain-related diagnoses included ICD-10-CM codes G89.29 and G89.4. CI, confidence interval; HR, hazard ratio; VDD, vitamin D deficiency. Hypocalcemia was defined as uncorrected total serum calcium <8.5 mg/dL.

**Table 8:** Association between chronic vitamin D deficiency and risk of incident fibromyalgia.

Outcome	Chronic VDD group (*n* = 116,156)	Control group (*n* = 116,156)	HR (95% CI)	*p* value
	Events (%)	Events (%)		
Fibromyalgia	1,151 (0.99)	540 (0.47)	1.97 (1.78–2.19)	<0.001
Chronic pain-related diagnosis	10,740 (9.25)	6,225 (5.36)	1.65 (1.60–1.70)	<0.001
Mortality	7,934 (6.83)	4,197 (3.61)	1.76 (1.70–1.83)	<0.001
Hyperparathyroidism	381 (0.33)	185 (0.16)	1.97 (1.65–2.35)	<0.001
Hypocalcemia	19,624 (16.90)	15,120 (13.02)	1.28 (1.26–1.31)	<0.001
Osteoporotic fracture	470 (0.40)	273 (0.24)	1.57 (1.35–1.82)	<0.001
Appendicitis	524 (0.45)	451 (0.39)	1.10 (0.97–1.24)	0.155
At least one healthcare visit during follow-up	106,355 (91.56)	108,518 (93.42)	0.89 (0.89–0.90)	<0.001

Chronic VDD was defined as two serum 25(OH)D measurements <20 ng/mL recorded within 1 year; chronic normal vitamin D status was defined as two serum 25(OH)D measurements ≥30 ng/mL recorded within 1 year. The index date was the date of the second qualifying measurement. Data are presented as number of events (%). CI, confidence interval; HR, hazard ratio; VDD, vitamin D deficiency. Hypocalcemia was defined as uncorrected total serum calcium <8.5 mg/dL.

### 3.6 Competing-risk analysis

A competing risk analysis was performed in the full unmatched cohort to estimate the cumulative incidence of fibromyalgia while treating mortality as a competing event (Table 9). The primary and competing-risk analyses were performed at different time points. Because the TriNetX network is continuously updated, the cohort sizes in this supplementary analysis differed from those reported for the primary analysis, although the same eligibility criteria were applied. The Aalen–Johansen cumulative incidence of fibromyalgia at the end of the follow-up window was 2.00% in the VDD cohort (*n* = 748,206) and 1.38% in the control cohort (*n* = 1,296,938), while the cumulative incidence of mortality was 10.86 and 10.29%, respectively. Despite the larger competing burden of mortality in the VDD cohort, the cumulative incidence of fibromyalgia remained higher than that in the control cohort, suggesting that the observed association was not attributable to the differential mortality between groups.

**Table 9:** Competing-risk analysis of incident fibromyalgia and mortality according to vitamin D status.

Cohort	Outcome	Crude event proportion, %	Aalen–Johansen cumulative incidence at end of time window
Vitamin D deficiency	Fibromyalgia	0.94%	2.00%
Vitamin D deficiency	Mortality	5.23%	10.86%
Normal vitamin D status	Fibromyalgia	0.62%	1.38%
Normal vitamin D status	Mortality	4.66%	10.29%

Cumulative incidence was estimated using the Aalen–Johansen estimator in the full unmatched cohort, with death treated as a competing event for incident fibromyalgia. VDD, vitamin D deficiency. The crude event proportion was calculated as the number of patients with the recorded outcome divided by the total cohort and did not account for censoring or competing events. The Aalen–Johansen estimate represents cumulative incidence at the end of the up-to-10-year analytic window while accounting for right censoring; death was treated as a competing event for incident fibromyalgia. Estimates were derived from the full unmatched cohort and should be interpreted descriptively.

## 4 Discussion

In this large propensity score–matched cohort study with a 1-year landmark design, low vitamin D status was associated with a higher long-term risk of incident fibromyalgia compared to normal vitamin D status (0.85% vs. 0.49%; absolute difference in observed event proportions, 0.36 percentage points; HR, 1.63). The observed relationship persisted consistently in sensitivity analyses, across subgroup strata, and in sequential multivariable Cox models. However, it was attenuated in Model I (HR, 1.35), in which the negative-control outcome of appendicitis also showed a modest association (HR, 1.13). This negative-control finding suggests that residual confounding, differential surveillance, or coding-related bias may remain in the contemporary-period analysis and warrants cautious interpretation of the corresponding fibromyalgia estimate. The severity and chronicity analyses also demonstrated associations with incident fibromyalgia under more stringent exposure definitions, including severe VDD and chronic VDD defined by two deficient 25(OH)D measurements within 1 year. These findings extend prior cross-sectional evidence by supporting a temporal association between VDD and subsequent fibromyalgia diagnosis in a large, multi-institutional electronic health record cohort. Although the relative association was notable, the absolute excess was modest at the individual level (0.36 percentage points). Given the high prevalence of VDD, even a small absolute increase could have population-health implications if the association were causal. Nevertheless, the observational design and potential residual confounding preclude causal conclusions, and these findings do not support vitamin D screening or supplementation for the prevention of fibromyalgia.

Most previous research on the vitamin D–fibromyalgia link has been cross-sectional in nature or has been conducted in patients with pre-existing fibromyalgia (18, 19, 21). Meta-analyses by Makrani et al. and Ismail et al. reported lower serum 25-hydroxyvitamin D [25(OH)D] concentrations among patients with fibromyalgia than among controls; however, such methodologies could not disentangle whether low vitamin D status was an antecedent of disease onset or simply a reflection of pre-existing illness (18, 19). A meta-analysis by Hsiao et al. (20) further found that hypovitaminosis D was associated with chronic widespread pain, with a pooled adjusted odds ratio of 1.41, providing directional support for a relationship between low vitamin D status and fibromyalgia-related pain phenotypes. McCabe et al. provided one of the few longitudinal assessments in this field by examining baseline vitamin D status and subsequent chronic widespread pain in middle-aged and elderly men, although the observed association was attenuated after accounting for adverse health factors such as obesity and depression (24). Randomized-trial findings have also been inconsistent. A double-blind, placebo-controlled trial found no improvement in pain intensity or fibromyalgia impact following vitamin D supplementation (25). A recent Mendelian randomization analysis similarly found no association of genetically predicted 25(OH)D concentrations with fibromyalgia or chronic widespread pain (26). Accordingly, our observational findings should not be interpreted as evidence supporting a causal effect, vitamin D screening, or supplementation for the prevention of fibromyalgia. Given that a recent systematic review highlighted both the possible pain-modulating effects of vitamin D and the continued uncertainty regarding its direct role in pain chronification (27), our study adds to this literature by specifically evaluating incident fibromyalgia, applying a landmark design to reduce reverse causation, matching patients on a broad set of pain-related, neuropsychiatric, metabolic, inflammatory, and medication-related covariates, and using prespecified control outcomes to assess internal validity.

Chronic pain–related diagnosis was included as a secondary outcome because fibromyalgia shares substantial clinical overlap with other persistent pain conditions (17, 28, 29), and diagnostic coding practices may vary across clinicians and healthcare systems. Widespread pain meeting clinical criteria for fibromyalgia may be coded under related diagnoses, such as chronic pain syndrome or other chronic pain. The consistent association between VDD and chronic pain–related diagnoses supports the robustness of the primary finding, although this broader outcome should be interpreted cautiously because it may capture heterogeneous pain conditions beyond fibromyalgia.

Several mechanisms may plausibly link VDD to pain amplification, although these pathways were not directly assessed in the present study. Vitamin D receptors are expressed in the nervous system and immune cells, and experimental evidence suggests that vitamin D may modulate neuroimmune signaling, microglial activation, oxidative stress, and pro-inflammatory cytokine release relevant to nociceptive processing (30–33). Current concepts of fibromyalgia emphasize altered pain processing, including central sensitization, neuroinflammatory and immune activation, impaired descending pain modulation, and altered peripheral sensory input (13, 14). Within this framework, VDD may be related to the risk of fibromyalgia through enhanced neuroinflammatory signaling, impaired endogenous pain inhibition, or increased peripheral nociceptive input from musculoskeletal and neuromuscular dysfunction. However, these mechanisms remain speculative in the present observational analysis and warrant confirmation in studies that incorporate mechanistic biomarkers.

The severity and chronicity analyses provided additional information regarding the association under more stringent exposure definitions. Severe and chronic VDD yielded larger effect estimates than the primary definition; however, these estimates were obtained from separately constructed and matched cohorts and should therefore be interpreted descriptively rather than as evidence of a formal dose–response trend. Importantly, the positive control outcome of secondary hyperparathyroidism also showed a stronger association in the severe deficiency analysis, consistent with the well-established metabolic consequence of VDD. This parallel pattern provides reassurance regarding the exposure definitions. Nevertheless, the larger effect estimates observed under the more stringent severity and chronicity definitions were derived from separate analyses and should be interpreted descriptively. They should not be considered evidence of a formal dose–response relationship or causality because residual confounding by health status, nutrition, physical activity, inflammatory burden, or healthcare-seeking behavior may still contribute to the observed associations.

Sex-stratified analyses showed that the association between VDD and incident fibromyalgia was present in both males and females, with no statistically significant difference between the subgroup-specific estimates. Although fibromyalgia is more frequently diagnosed in women (34), our findings suggest that the association between low vitamin D status and fibromyalgia risk is not restricted to one sex. This observation is consistent with the European Male Aging Study, which reported an association between low vitamin D status and chronic widespread pain in men (24). In age-stratified analyses, the association was stronger among adults aged > 65 years. The larger association observed in adults aged >65 years may reflect the convergence of several vulnerability factors in older adults, including a higher prevalence of sustained VDD, greater comorbidity burden, age-related decline in descending pain inhibitory capacity, and increased neuroinflammatory burden (35–37). However, age-related differences in diagnostic coding, healthcare contact frequency, pain reporting, and referral patterns cannot be excluded. Thus, this subgroup finding should be interpreted cautiously and considered as hypothesis-generating.

Competing risk analysis also provides a complementary perspective. In the full unmatched cohort, the Aalen–Johansen cumulative incidence of fibromyalgia was higher in the VDD cohort than in the normal vitamin D cohort, despite a slightly higher cumulative incidence of mortality. This finding suggests that the higher observed cumulative incidence of fibromyalgia is unlikely to be explained solely by the competing risk of death. However, because this analysis was performed in the full unmatched cohort, it should be interpreted as supportive descriptive evidence rather than as a replacement for the matched cause-specific Cox analysis. In addition, both cohorts underwent baseline 25(OH)D testing, and the VDD cohort did not have greater overall follow-up contact: at least one healthcare encounter was recorded in 91.17% of the VDD cohort and 91.75% of the control cohort (HR, 0.90). The association with incident fibromyalgia also remained when the analysis was restricted to patients with at least one follow-up encounter (Model III: HR, 1.54). These findings argue against the simplest form of surveillance bias based solely on the presence of any follow-up contact. However, the clinical indications for vitamin D testing were unavailable, and the binary healthcare-utilization measure could not capture visit frequency, symptom burden, specialty referral, or pain-related diagnostic intensity; therefore, differential detection of fibromyalgia cannot be excluded.

This study has several limitations. First, the observational design precludes causal inference, and residual confounding from unmeasured factors, including sunlight exposure, physical activity, dietary intake, socioeconomic status, psychosocial stress, sleep quality, catastrophizing, and physical deconditioning, cannot be excluded from the study. Second, although propensity score matching and sequential multivariable adjustment balanced many measured covariates, low vitamin D status may function primarily as a marker of poorer overall health status and related vulnerabilities, including inflammation, frailty, obesity, and differences in healthcare-seeking behavior, rather than as a direct causal determinant of fibromyalgia. Mortality was not intended as corroborative evidence for the VDD–fibromyalgia association; rather, its persistent elevation after matching may indicate residual differences in frailty, immobility, inflammatory burden, or overall health status. The relatively low recorded prevalence of depressive and sleep disorders may similarly reflect incomplete coding and limited control of these potential confounder. This possibility limits the causal interpretation of the observed association. Third, the primary exposure definition relied on a single baseline 25(OH)D measurement, which may not fully represent long-term vitamin D status and may therefore result in exposure misclassification when vitamin D levels change over time. Although the chronic deficiency analysis requiring repeated low measurements partially reduced this concern, post-index vitamin D supplementation, supplementation dose and adherence, and subsequent biochemical correction of deficiency were not assessed as time-varying factors. Correction of deficiency during follow-up could have resulted in exposure crossover and attenuated the observed association, whereas differential prescribing, adherence, or correction between groups could have biased the estimates in either direction. Because the calendar month or season of the index measurement was unavailable in the aggregated analytic output, seasonality could not be assessed. Seasonal variation may therefore have shifted some patients across the prespecified vitamin D thresholds and contributed to exposure misclassification. Fourth, fibromyalgia was ascertained using a single recorded ICD-10-CM M79.7 code without confirmation through repeated coding or independent validation against clinical diagnostic criteria, which may have resulted in outcome misclassification because fibromyalgia is frequently underdiagnosed and diagnostic and coding practices vary across clinicians and healthcare systems. If nondifferential, such outcome misclassification would likely attenuate the observed association toward the null; however, differential surveillance, referral, or coding practices between groups could have biased the estimates in either direction. Fifth, outcome-specific total person-time at risk was unavailable in the aggregated analytic output, preventing reliable calculation of incidence rates per 1,000 person-years. Consequently, absolute risks were described using observed event proportions, which should be interpreted alongside the reported follow-up durations. Finally, although the landmark design reduced reverse causation, it could not fully exclude the possibility that subclinical pain, reduced activity, or early health deterioration preceded both VDD and subsequent fibromyalgia diagnosis.

## 5 Conclusion

Low vitamin D status was associated with a higher long-term risk of incident fibromyalgia in this large propensity score–matched cohort study. Associations were also observed under the severe and chronic deficiency definitions and remained generally consistent across subgroup, sensitivity, multivariable, and competing-risk analyses. These findings support a temporal association between low vitamin D status and subsequent fibromyalgia diagnosis; however, they should not be interpreted as evidence of causality. Prospective studies with repeated vitamin D measurements, detailed pain phenotyping, lifestyle data, and inflammatory biomarkers are needed to clarify the temporal and biological relationships between low vitamin D status and fibromyalgia development.

## Data Availability

The raw data supporting the conclusions of this article will be made available by the authors, without undue reservation.
