# Supplementary material for: Low vitamin D status and long-term risk of incident fibromyalgia: a propensity score–matched cohort study
Source: Front Nutr. 2026 Aug 24;13:1889243. doi: 10.3389/fnut.2026.1889243 (PMC13547082; doi:10.3389/fnut.2026.1889243)
Supplement: Supplementary file 1 [file Table_1.DOCX]

**Supplemental Table 1**

**Complete code lists and query definitions for the TriNetX retrospective cohort analysis**

| **Query element** | **Specification** |
| --- | --- |
| **Data source** | TriNetX Global Collaborative Network |
| **Study data-extraction date** | May 9, 2026 |
| **Primary query names** | @@ VDD v1 and @@ Control v1 |
| **Study entry period** | January 1, 2010 through December 31, 2024 |

Codes, labels, thresholds, and temporal relations are reproduced from the supplied TriNetX Compare Outcomes export and reconciled with the revised manuscript. Codes without an explicit namespace in the export are identified as “TriNetX export code.”

## Panel A. Cohort construction and analytic query logic

| **Query element** | **Specification** |
| --- | --- |
| **Population** | Adults aged ≥18 years with an eligible serum 25-hydroxyvitamin D measurement. |
| **Exposure cohort** | Low vitamin D status: first qualifying calcidiol or calcidiol/ercalcidiol result <20 ng/mL (TriNetX operational threshold ≤19.90 ng/mL). |
| **Comparator cohort** | Normal vitamin D status: first qualifying calcidiol or calcidiol/ercalcidiol result ≥30 ng/mL. |
| **Index date** | Date of the first laboratory result meeting the exposure or comparator definition; index events were restricted to the study entry period. |
| **Intermediate values** | Values of 20.0–29.9 ng/mL did not qualify a patient for either cohort. |
| **Exposure-specific look-back** | During the 5 years and 1 day before index, the low-vitamin-D cohort could not have a value ≥20.00 ng/mL, and the normal-vitamin-D cohort could not have a value ≤29.90 ng/mL. |
| **Baseline encounter requirement** | At least one recorded visit within 3 years and 1 day before index and at least one additional visit between 5 years and 3 years before index (two non-overlapping look-back intervals). |
| **Pre-index exclusions** | Renal failure/dialysis, selected psychiatric and pain-confounding conditions, bariatric surgery, osteomalacia, malabsorptive disorders, and hepatic fibrosis/cirrhosis were excluded when recorded before index, using the codes in Panel B. |
| **Landmark exclusions** | Patients with fibromyalgia, other chronic pain, chronic pain syndrome, osteoporosis with current pathological fracture, death, or R99 before or through 1 year after index were excluded, as defined in the exported query. |
| **Outcome window** | Outcomes were evaluated from day 365 through day 3,650 after index (up to 10 years after index). |
| **Outcome settings** | The supplied export specified risk and Kaplan–Meier analyses and retained patients with the outcome before the analytic time window; incident ascertainment for the primary pain outcomes was implemented through the cohort and landmark exclusions above. |
| **Matching** | 1:1 greedy nearest-neighbor propensity-score matching with a caliper of 0.1 pooled standard deviations; the covariates and codes are listed in Panel C. |

## Panel B. Exposure, eligibility, exclusion, and landmark code list

| **Role/domain** | **Concept or variable** | **Vocabulary and code** | **Exact query use or restriction** |
| --- | --- | --- | --- |
| Eligibility | Age | Demographics: Age | Most recent occurrence ≥18 years. |
| Exposure laboratory | Calcidiol [Mass/volume] in serum or plasma | TNX:9034 | Low vitamin D: ≤19.90 ng/mL; normal vitamin D: ≥30.00 ng/mL. |
|  | Calcidiol + ercalcidiol [Mass/volume] in serum, plasma, or blood | TNX:LG25965-1 | Low vitamin D: ≤19.90 ng/mL; normal vitamin D: ≥30.00 ng/mL. |
| Encounter | Visit | TNX:Visit | Used for the two baseline encounter look-back requirements. |
| Incident/landmark exclusion | Osteoporosis with current pathological fracture | UMLS:ICD10CM:M80 | Excluded before or through 1 year after index. |
|  | Fibromyalgia | UMLS:ICD10CM:M79.7 | Excluded before or through 1 year after index. |
|  | Other chronic pain | UMLS:ICD10CM:G89.29 | Excluded before or through 1 year after index. |
|  | Chronic pain syndrome | UMLS:ICD10CM:G89.4 | Excluded before or through 1 year after index. |
| Renal exclusion | Chronic kidney disease, stage 4 (severe) | UMLS:ICD10CM:N18.4 | Excluded before index. |
|  | Chronic kidney disease, stage 5 | UMLS:ICD10CM:N18.5 | Excluded before index. |
|  | End-stage renal disease | UMLS:ICD10CM:N18.6 | Excluded before index. |
|  | Dependence on renal dialysis | UMLS:ICD10CM:Z99.2 | Excluded before index. |
| Psychiatric exclusion | Bipolar disorder | UMLS:ICD10CM:F31 | Excluded before index. |
|  | Schizophrenia, schizotypal, delusional, and other non-mood psychotic disorders | UMLS:ICD10CM:F20–F29 | Excluded before index. |
| Bariatric exclusion | Bariatric surgery status | UMLS:ICD10CM:Z98.84 | Excluded before index. |
|  | Laparoscopic sleeve gastrectomy | UMLS:CPT:43775 | Excluded before index. |
|  | Laparoscopic bariatric surgery procedures | UMLS:CPT:1007386 | Excluded before index; concept reproduced exactly as exported. |
|  | Bariatric surgery procedures | UMLS:CPT:1007385 | Excluded before index; concept reproduced exactly as exported. |
|  | Laparoscopic gastric bypass with Roux-en-Y gastroenterostomy | UMLS:CPT:43644 | Excluded before index. |
| Pain-confounder exclusion | Opioid-related disorders | UMLS:ICD10CM:F11 | Excluded before index. |
|  | Rheumatoid arthritis with rheumatoid factor | UMLS:ICD10CM:M05 | Excluded before index. |
|  | Other rheumatoid arthritis | UMLS:ICD10CM:M06 | Excluded before index. |
|  | Systemic lupus erythematosus | UMLS:ICD10CM:M32 | Excluded before index. |
|  | Dermatopolymyositis | UMLS:ICD10CM:M33 | Excluded before index. |
| Metabolic/absorption exclusion | Adult osteomalacia | UMLS:ICD10CM:M83 | Excluded before index. |
|  | Celiac disease | UMLS:ICD10CM:K90.0 | Excluded before index. |
|  | Crohn disease | UMLS:ICD10CM:K50 | Excluded before index. |
|  | Ulcerative colitis | UMLS:ICD10CM:K51 | Excluded before index. |
|  | Postsurgical malabsorption, not elsewhere classified | UMLS:ICD10CM:K91.2 | Excluded before index. |
|  | Fibrosis and cirrhosis of liver | UMLS:ICD10CM:K74 | Excluded before index. |
| Landmark mortality exclusion | Deceased status | Demographics: Deceased | Excluded before or through 1 year after index. |
|  | Ill-defined and unknown cause of mortality | UMLS:ICD10CM:R99 | Excluded before or through 1 year after index. |

## Panel C. Propensity-score matching covariates and codes

| **Role/domain** | **Concept or variable** | **Vocabulary and code** | **Exact query use or restriction** |
| --- | --- | --- | --- |
| Demographic | Age at index | TriNetX export code: AI | Continuous age at index. |
|  | Female | TriNetX export code: F | Binary demographic covariate. |
|  | White | TriNetX export code: 2106-3 | Race category. |
|  | Black or African American | TriNetX export code: 2054-5 | Race category. |
|  | Hispanic or Latino | TriNetX export code: 2135-2 | Ethnicity category. |
|  | Asian | TriNetX export code: 2028-9 | Race category. |
| Diagnosis | Essential (primary) hypertension | ICD-10-CM: I10 | Baseline covariate. |
|  | Diabetes mellitus | ICD-10-CM: E08–E13 | Baseline covariate. |
|  | Nicotine dependence | ICD-10-CM: F17 | Baseline covariate. |
|  | Ischemic heart diseases | ICD-10-CM: I20–I25 | Baseline covariate. |
|  | Chronic kidney disease | ICD-10-CM: N18 | Baseline covariate. |
|  | Alcohol-related disorders | ICD-10-CM: F10 | Baseline covariate. |
|  | Cerebrovascular diseases | ICD-10-CM: I60–I69 | Baseline covariate. |
|  | Other chronic obstructive pulmonary disease | ICD-10-CM: J44 | Baseline covariate. |
|  | Malnutrition | ICD-10-CM: E40–E46 | Baseline covariate. |
|  | Sleep disorders | ICD-10-CM: G47 | Baseline covariate. |
|  | Heart failure | ICD-10-CM: I50 | Baseline covariate. |
|  | Anxiety, dissociative, stress-related, somatoform, and other nonpsychotic mental disorders | ICD-10-CM: F40–F48 | Baseline covariate. |
|  | COVID-19 | ICD-10-CM: U07.1 | Baseline covariate. |
|  | Diseases of liver | ICD-10-CM: K70–K77 | Baseline covariate. |
|  | Encounter for general adult medical examination | ICD-10-CM: Z00.0 | Baseline healthcare-utilization covariate. |
|  | Disorders of thyroid gland | ICD-10-CM: E00–E07 | Baseline covariate. |
|  | Dorsalgia | ICD-10-CM: M54 | Baseline covariate. |
|  | Pain, not elsewhere classified | ICD-10-CM: G89 | Baseline covariate. |
|  | Iron deficiency anemia | ICD-10-CM: D50 | Baseline covariate. |
|  | Other nutritional deficiencies | ICD-10-CM: E50–E64 | Baseline covariate. |
|  | Neoplasms | ICD-10-CM: C00–D49 | Baseline covariate. |
|  | Reduced mobility | ICD-10-CM: Z74.0 | Baseline covariate. |
|  | Systemic connective tissue disorders | ICD-10-CM: M30–M36 | Baseline covariate. |
|  | Disorders of lipoprotein metabolism and other lipidemias | ICD-10-CM: E78 | Baseline covariate. |
|  | Osteoarthritis | ICD-10-CM: M15–M19 | Baseline covariate. |
|  | Chronic fatigue, unspecified | ICD-10-CM: R53.82 | Baseline covariate. |
|  | Major depressive disorder, recurrent | ICD-10-CM: F33 | Baseline covariate. |
|  | Migraine | ICD-10-CM: G43 | Baseline covariate. |
| Medication | Benzodiazepine derivative sedatives/hypnotics | TriNetX export code: CN302 | Baseline medication exposure. |
|  | Insulins and analogues | ATC/TriNetX export code: A10A | Baseline medication exposure. |
|  | Blood glucose-lowering drugs, excluding insulins | ATC/TriNetX export code: A10B | Baseline medication exposure. |
|  | Vitamin D | TriNetX export code: VT500 | Baseline medication exposure. |
|  | Corticosteroids for systemic use | ATC/TriNetX export code: H02 | Baseline medication exposure. |
|  | Opioid analgesics | TriNetX export code: CN101 | Baseline medication exposure. |
|  | Antidepressants | ATC/TriNetX export code: N06A | Baseline medication exposure. |
|  | Gabapentinoids | ATC/TriNetX export code: N02BF | Baseline medication exposure. |
| Laboratory | Albumin [Mass/volume] in serum, plasma, or blood | TriNetX export code: 9045 | Prespecified category: ≤3.5 g/dL; continuous values also shown in the export. |
|  | Creatinine-based eGFR (CKD-EPI 2021)/1.73 m² | TriNetX export code: 98979-8 | Prespecified category: ≤60 mL/min/1.73 m²; continuous values also shown in the export. |
|  | Hemoglobin A1c/total hemoglobin in blood | TriNetX export code: 9037 | Prespecified category: ≥9%; continuous values also shown in the export. |
|  | C-reactive protein [Mass/volume] in serum, plasma, or blood | TriNetX export code: 9063 | Prespecified category: ≥10 mg/L; continuous values also shown in the export. |
|  | Hemoglobin [Mass/volume] in blood | TriNetX export code: 9014 | Prespecified category: ≥12 g/dL; continuous values also shown in the export. |
|  | Body mass index | TriNetX export code: 9083 | Prespecified category: ≥30 kg/m²; continuous values also shown in the export. |

## Panel D. Outcome definitions and codes

| **Role/domain** | **Concept or variable** | **Vocabulary and code** | **Exact query use or restriction** |
| --- | --- | --- | --- |
| Primary outcome | Fibromyalgia | UMLS:ICD10CM:M79.7 | Any qualifying diagnosis in the outcome window. |
| Secondary outcome | Other chronic pain | UMLS:ICD10CM:G89.29 | Combined with G89.4 as the chronic pain-related diagnosis outcome. |
|  | Chronic pain syndrome | UMLS:ICD10CM:G89.4 | Combined with G89.29 as the chronic pain-related diagnosis outcome. |
| Contextual outcome | Deceased status | Demographics: Deceased | Combined with R99 for all-cause mortality. |
|  | Ill-defined and unknown cause of mortality | UMLS:ICD10CM:R99 | Combined with the Deceased demographic status. |
| Positive control | Osteoporosis with current pathological fracture | UMLS:ICD10CM:M80 | Any qualifying diagnosis in the outcome window. |
|  | Secondary hyperparathyroidism, not elsewhere classified | UMLS:ICD10CM:E21.1 | Any qualifying diagnosis in the outcome window. |
|  | Hypocalcemia | TNX:9022 | Most recent uncorrected total calcium result ≤8.50 mg/dL in serum, plasma, or blood. |
| Negative control | Accute appendix | UMLS:ICD10CM:K35 | Any qualifying diagnosis in the outcome window. |
| Healthcare utilization | At least one visit | TNX:Visit | Binary indicator of ≥1 visit in the outcome window. |
| Additional exported outcome | Subsequent low vitamin D result | TNX:9034 | Most recent calcidiol result ≤19.90 ng/mL in the outcome window. |
|  | Subsequent low vitamin D result | TNX:LG25965-1 | Most recent calcidiol + ercalcidiol result ≤19.90 ng/mL in the outcome window. |
|  | Gabapentinoids | NLM:ATC:N02BF | Combined medication outcome in the supplied export. |
|  | Pregabalin | NLM:RXNORM:187832 | Combined medication outcome in the supplied export. |
|  | Duloxetine | NLM:RXNORM:72625 | Combined medication outcome in the supplied export. |
|  | Milnacipran | NLM:RXNORM:588250 | Combined medication outcome in the supplied export. |

## Panel E. Analysis-specific query modifications

| **Role/domain** | **Concept or variable** | **Vocabulary and code** | **Exact query use or restriction** |
| --- | --- | --- | --- |
| Primary analysis | Low vitamin D vs normal vitamin D | TNX:9034; TNX:LG25965-1 | <20 ng/mL (operationally ≤19.90) vs ≥30 ng/mL; 2010–2024; base criteria in Panels A–D. |
| Sensitivity Model I | Recent-data restriction | No additional code | Restricted the study entry period to 2016–2024; all other definitions unchanged. |
| Sensitivity Model II | Survivor restriction | Demographics: Deceased; UMLS:ICD10CM:R99 | Excluded patients who died during follow-up; all other definitions unchanged. |
| Sensitivity Model III | Follow-up encounter restriction | TNX:Visit | Required at least one recorded healthcare encounter during follow-up. |
| Sex subgroup | Male vs female | Demographic sex categories | Each stratum was queried and matched separately. |
| Age subgroup | 18–65 vs >65 years | Demographics: Age | Each stratum was queried and matched separately. |
| Severity analysis | Severe vitamin D deficiency | TNX:9034; TNX:LG25965-1 | <10 ng/mL vs normal vitamin D status ≥30 ng/mL; other base criteria retained. |
| Chronicity analysis | Repeated vitamin D measurements | TNX:9034; TNX:LG25965-1 | Two values <20 ng/mL within 1 year vs two values ≥30 ng/mL within 1 year; index date was the second qualifying measurement. |

Abbreviations: ATC, Anatomical Therapeutic Chemical classification; CPT, Current Procedural Terminology; eGFR, estimated glomerular filtration rate; HbA1c, hemoglobin A1c; HCO, healthcare organization; ICD-10-CM, International Classification of Diseases, Tenth Revision, Clinical Modification; NLM, U.S. National Library of Medicine; RxNorm, normalized drug vocabulary; TNX, TriNetX; UMLS, Unified Medical Language System.

Category codes and ranges are reported at the level shown in the supplied TriNetX export. The supplied primary export queried 171 HCOs in the Global Collaborative Network. Separate analysis-specific queries retained the same base code set unless a modification is stated in Panel E.
