# Supplementary material for: Low vitamin D status and long-term risk of incident fibromyalgia: a propensity score–matched cohort study
Source: Front Nutr. 2026 Aug 24;13:1889243. doi: 10.3389/fnut.2026.1889243 (PMC13547082; doi:10.3389/fnut.2026.1889243)
Supplement: Supplementary file 2 [file Table_2.DOCX]

**Supplemental Table 2**. Categorized distribution and missingness of laboratory covariates after propensity score matching

| **Laboratory covariate** | **Category** | **VDD cohort (n = 561,418), n (%)** | **Control cohort (n = 561,418), n (%)** |
| --- | --- | --- | --- |
| **Hemoglobin** | ≥12 g/dL | 360,806 (64.27%) | 367,562 (65.47%) |
|  | <12 g/dL | 32,985 (5.88%) | 23,012 (4.10%) |
|  | Not measured | 167,627 (29.86%) | 170,844 (30.43%) |
| **Estimated glomerular filtration rate** | >60 mL/min/1.73 m² | 301,722 (53.74%) | 284,976 (50.76%) |
|  | ≤60 mL/min/1.73 m² | 70,674 (12.59%) | 74,043 (13.19%) |
|  | Not measured | 189,022 (33.67%) | 202,399 (36.05%) |
| **Serum albumin** | >3.5 g/dL | 253,274 (45.11%) | 257,577 (45.88%) |
|  | ≤3.5 g/dL | 62,935 (11.21%) | 64,659 (11.52%) |
|  | Not measured | 245,209 (43.68%) | 239,182 (42.60%) |
| **Hemoglobin A1c** | <9% | 161,136 (28.70%) | 152,367 (27.14%) |
|  | ≥9% | 19,775 (3.52%) | 19,437 (3.46%) |
|  | Not measured | 380,507 (67.78%) | 389,614 (69.40%) |
| **C-reactive protein** | <10 mg/L | 47,604 (8.48%) | 39,856 (7.10%) |
|  | ≥10 mg/L | 30,148 (5.37%) | 29,433 (5.24%) |
|  | Not measured | 483,666 (86.15%) | 492,129 (87.66%) |

Values are n (%). Percentages were calculated using the full matched cohort as the denominator. VDD, vitamin D deficiency.
